# The gut within: reframing the coral gastrovascular cavity as a discrete anaerobic microbial ecosystem

**DOI:** 10.1128/aem.01430-26

**Published:** 2026-10-05

**Authors:** Claudia Pogoreutz

**Affiliations:** 1 UAR3278, PSL University Paris: CRIOBE EPHE-CNRS-UPVD, Université de Perpignan Via Domitia 27074, Perpignan, France; Michigan State University, East Lansing, Michigan, USA

**Keywords:** coral holobiont, microenvironment, gradient, anaerobic metabolism, denitrification, fermentation, microsensors

## Abstract

Coral holobionts are typically studied at colony or fragment scale, erasing the spatial context of the microenvironments which microbial life occupies. Recent microsensor work (Q. Zhang, E. Bollati, and M. Kühl, Appl Environ Microbiol 92:e02441-25, 2026, https://doi.org/10.1128/aem.02441-25) shows the coral gastrovascular cavity (GVC) sustains active anaerobic metabolism, reflecting patterns of symbiotic state and heterotrophic feeding. These findings recast the GVC as a discrete, gut-like ecosystem with its own redox regime, substrate supply, and bacterial community, calling for spatially explicit approaches in coral research to resolve metabolism at microscopic scales.

## COMMENTARY

Resembling delicate plants or unassuming boulders, reef-building corals are among the most intensively studied animal–microbe symbioses in the sea. Yet most of what we know about their associated microbes comes from measurements made on entire colonies or fragments. While this scale is convenient for experimental and field work, this approach eliminates spatial context, as it averages across compartments that differ enormously in their physicochemical and biological properties. These compartments include the tissues, the surface mucus layer at the epithelium–ocean interface, the porous aragonite skeleton beneath, and the gastrovascular cavity (GVC), a fluid-filled space where prey is digested, where algal symbionts are taken up or expelled, and through which resources are shared among individual coral polyps (1, 2). Treating these distinct compartments as one obscures precisely the physicochemical gradients that structure microbial activity. A recent study by Zhang and colleagues brings the GVC into focus, making a compelling case that this understudied coral compartment deserves—and needs—to be understood as a distinct system (3).

Using microsensors with tip diameters of 50 µm, Zhang et al. profiled oxygen, pH, hydrogen, nitric oxide, and nitrous oxide directly within the GVC of individual polyps of the corals *Astraeosmilia curvata* and *Goniastrea* sp. These measurements reveal a compartment that swings between chemical extremes over the diel cycle. Under light, the GVC of healthy corals is rendered hyperoxic due to the photosynthetic activity of the algal endosymbionts. Yet in darkness—in the absence of oxygenic photosynthesis—the GVC becomes hypoxic and shifts toward acidic conditions, with pH dropping by more than one unit. Against this hypoxic backdrop, the authors detected hydrogen, nitric oxide, and nitrous oxide—chemical signatures of fermentation and denitrification—providing evidence that anaerobic microbial metabolism is not merely possible in the GVC but is indeed happening (3). What makes this microsensing view particularly fascinating is that the anaerobic signal dynamically responds to two major variables which define the physiological state of coral: symbiotic state and heterotrophic feeding.

**Fig 1:**
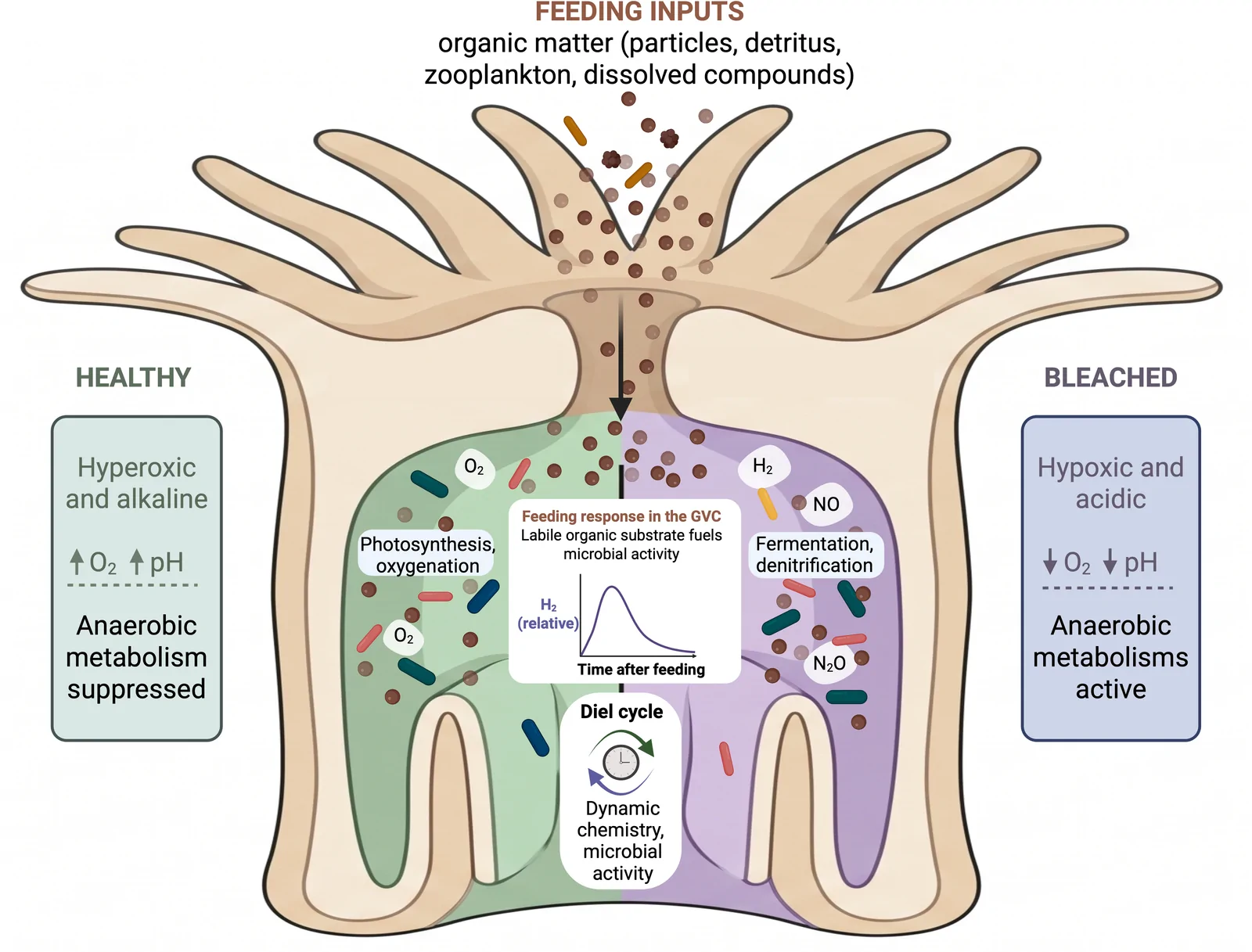
Proposed daytime interactions between symbiotic state, heterotrophic feeding activity, and anaerobic microbial processes in the coral gastrovascular cavity (GVC). In healthy coral, photosynthesis by the algal endosymbionts shapes diurnal physicochemical cycles, thereby driving patterns of anaerobic activity. In bleached corals, which have lost their endosymbionts, anaerobic microbes are released from the constraints of daytime oxygenation, permitting sustained anaerobic activity. Heterotrophic feeding pulses result in rapid spikes of anaerobic activity, which decline as food particles are increasingly exhausted and/or as H_2_ is consumed by microbes, adding to the complex interplay of factors that may shape chemical fluctuations in the GVC.

Symbiotic state in corals is defined by whether algal endosymbionts are present and active in the tissues or not. These algal symbionts are important nutritional intracellular partners, which have shaped the ecological and evolutionary success of corals—and thereby reef formation as we know it today—over millions of years (4). In bleached polyps, which have lost these photosynthetic partners, the GVC remained hypoxic and acidic across the entire diel cycle, and signatures consistent with hydrogen production and denitrification persisted, even in the light. This suggests that in healthy corals, anaerobic metabolism is strongly suppressed during daytime due to photosynthetic activity but remains continuously active once that oxygen source is gone. Similarly, heterotrophic feeding has a profound impact on anaerobic microbial processes in the GVC. Following feeding, hydrogen concentrations in the GVC rose sharply—within minutes in *Goniastrea* corals—and then declined over the subsequent hours to a day, consistent with changing substrate availability and/or microbial consumption of H_2_ (3). The GVC, thus, seemingly behaves as a compartment whose microbial activity is modulated by host feeding activity, as one would expect of a functional digestive space (Fig. 1).

This is where the analogy to an animal gut becomes more than mere rhetoric. The GVC concentrates dissolved inorganic nitrogen to levels far above the surrounding seawater, receives pulses of organic matter through feeding, and generates steep oxygen gradients that permit anaerobic microbial niches—a combination of features that makes both vertebrate and invertebrate guts veritable hotspots of fermentation and nitrogen transformation (5, 6). Zhang et al. correlated their microsensing approach with 16S rRNA gene sequencing and found that bacterial community composition shifts along these gradients: polyps with elevated nitric oxide were enriched in putative denitrifiers, such as *Alcanivoracaceae* and *Xanthobacteraceae*, aligning bacterial identity with chemical environment. The picture that emerges is that of a spatially defined ecosystem in which local redox conditions may help structure microbial community composition and metabolic potential (3).

Reframing the GVC this way also has consequences for how we interpret bleaching. Although Zhang et al. cannot disentangle the effects of bleaching from other stressors associated with transport of their experimental corals, their compartment-scale view adds a critical new dimension to our understanding of coral bleaching. Coral bleaching is understood as the collapse of the cnidarian–algal symbiosis and the resulting energy deficit for the host, which forces dependence on heterotrophic feeding (7, 8). Here, Zhang et al. show that this altered physiological state is accompanied by a profoundly altered internal chemical landscape. Specifically, rather than cycling daily between oxic and hypoxic states, the GVC of bleached coral remained persistently oxygen-depleted. Such a shift may favor sustained anaerobic metabolism and, as Zhang et al. observed, shifts in the GVC-associated bacterial community, including an increase in *Staphylococcaceae*, a family containing opportunistic taxa (3). This raises the possibility that if the GVC becomes a more permissive niche for opportunists as symbiosis fails, compartment-scale processes may help explain the heightened disease susceptibility of bleached and heat-stressed corals—a link that whole-colony or fragment measurements alone are poorly positioned to detect, and that creates an exciting avenue for future research integrating spatial and functional approaches.

The work presented by Zhang et al. converges with a growing recognition that the coral holobiont is not spatially uniform; rather, it is becoming increasingly clear that coral compartments must be studied at ecologically meaningful scales to disentangle the likely complex relationship between holobiont state, microbes, and their activity within the GVC (9, 10). Microsensor profiling and micro-optode particle imaging have already begun to reshape our understanding of processes on the coral tissue surface and within the skeleton (10–13); extending them into the GVC—paired with a microvolume sampling approach for bacterial community profiling—moves us toward a compartment-by-compartment mapping of coral microenvironments (3, 14). This effort raises questions that only a compartment-scale approach can pose. How do feeding frequency and food composition modulate nutrient cycling? Can denitrification in the GVC contribute to maintaining the nitrogen-limited state of the holobiont on which the symbiosis ultimately depends? Do internal chemical processes scale up to whole-colony and even reef-level biogeochemistry?

Meeting these challenges will require moving beyond bulk sampling efforts toward tools that resolve microbial identity, activity, and chemistry in their spatial context. Several such approaches are already established in coral tissues, yet some still await broader application to the GVC. Correlated high-resolution imaging, NanoSIMS, and fluorescence *in situ* hybridization, for instance, have been used to localize hotspots of carbon or nitrogen assimilation within coral tissues and trace their isotopically labeled products in the intact symbiosis (15, 16). Tracing isotopic fate following a ^15^N-labeled feeding pulse could reveal which microbial residents in the GVC assimilate feeding-derived nitrogen and whether taxa with denitrifying potential participate in its assimilation, providing a direct link between host nutrition and microbial nitrogen cycling. Spatial metabolomics by matrix-assisted laser desorption/ionization (MALDI) mass spectrometry imaging on cryosectioned hard corals can resolve metabolites in the tissue lining the gastrovascular cavity (17) and could map the fermentation products and organic acids inferred by microsensing, showing where in the GVC they accumulate. Finally, techniques honed in gut or soil microbiology could be adapted to coral: for instance, bioorthogonal non-canonical amino acid tagging (BONCAT) fluorescently marks translationally active cells and, coupled with taxonomic identification, can resolve active bacterial subpopulations in complex host-associated communities (18). Fine-tuning this technique for coral could help answer not merely who is present in the GVC, but who is doing what. Together, such spatially explicit, activity-resolved approaches would constitute a natural next step in reading the inner life of coral.

Recognizing the GVC as a discrete anaerobic ecosystem ultimately reframes our view of a familiar, yet still poorly understood structure within the coral. The GVC is not simply where food is digested. Rather, it appears to be the site of an intimate interplay of oxygen gradients, substrate pulses, and microbial metabolism, which transforms nutrients in ways that may bear directly on holobiont function, health, and stress resilience. As coral reefs face intensifying pressure from the Anthropocene, understanding how these “ecosystems within” respond and how they are reconfigured by bleaching will be an essential part of understanding the responses of corals to global environmental change. In this context, the contribution of Zhang et al. lies not merely in the direct evidence of anaerobic metabolism it provides, but it reinforces the emerging view of coral as a collection of distinct microbial ecosystems, each worth understanding in its own right.
